# Multiple steps control immunity during the intracellular accommodation of rhizobia

**DOI:** 10.1093/jxb/eru545

**Published:** 2015-02-14

**Authors:** Fathi Berrabah, Pascal Ratet, Benjamin Gourion

**Affiliations:** Institut des Sciences du Végétal, CNRS, Saclay Plant Sciences, Avenue de la Terrasse, 91198 Gif sur Yvette, France

**Keywords:** *bacA*, *CRK*, *DNF2*, innate immunity, *nifA*, *nifH*, nitrogen fixation.

## Abstract

*Medicago truncatula* belongs to the legume family and forms symbiotic associations with nitrogen fixing bacteria, the rhizobia. During these interactions, the plants develop root nodules in which bacteria invade the plant cells and fix nitrogen for the benefit of the plant. Despite massive infection, legume nodules do not develop visible defence reactions, suggesting a special immune status of these organs. Some factors influencing rhizobium maintenance within the plant cells have been previously identified, such as the *M. truncatula* NCR peptides whose toxic effects are reduced by the bacterial protein BacA. In addition, *DNF2*, *SymCRK*, and *RSD* are *M. truncatula* genes required to avoid rhizobial death within the symbiotic cells. *DNF2* and *SymCRK* are essential to prevent defence-like reactions in nodules after bacteria internalization into the symbiotic cells. Herein, we used a combination of genetics, histology and molecular biology approaches to investigate the relationship between the factors preventing bacterial death in the nodule cells. We show that the *RSD* gene is also required to repress plant defences in nodules. Upon inoculation with the *bacA* mutant, defence responses are observed only in the *dnf2* mutant and not in the *symCRK* and *rsd* mutants. In addition, our data suggest that lack of nitrogen fixation by the bacterial partner triggers bacterial death in nodule cells after bacteroid differentiation. Together our data indicate that, after internalization, at least four independent mechanisms prevent bacterial death in the plant cell. These mechanisms involve successively: DNF2, BacA, SymCRK/RSD and bacterial ability to fix nitrogen.

## Highlight

Persistence of intracellular rhizobia in legumes involves plant and bacterial genes. Here we show that *DNF2*, *bacA*, *SymCRK*/*RSD* and *nifA/nifH* successively prevent bacterial death during the symbiotic process.

## Introduction

Legume plants can be cultivated without application of nitrogen fertilizers on soil poor in nitrogen. This capacity is the result of the associations with soil bacteria, referred to as rhizobia, that fix nitrogen for the benefit of the legume (for recent review, see Udvardi and Poole, 2013). These symbioses are efficient thanks to the spectacular bacterial density within the symbiotic organ, the nodule. Despite the massive and chronic colonization by bacteria, legume nodules do not develop defences. As an illustration of this, expression of defence-related genes is drastically reduced in nodule infected cells as compared to non-infected cells (Limpens *et al.*, 2013). A growing body of recent publications indicates that (the control of) plant innate immunity plays a major role during the early steps of rhizobium/legume interactions (Jones *et al.*, 2008; Yang *et al.*, 2010; Gough and Jacquet, 2013; Liang *et al.*, 2013; Lopez-Gomez *et al.*, 2012; Okazaki *et al.*, 2013).

However, the control of legume immunity is not only critical during the first steps of the symbiotic process but is also required once the nodule is formed when bacteria are released into the plant cells. In *Medicago truncatula* Gaertn., one of the favourite model legumes, two genes required to repress defence-like reactions into the nodules have been identified recently: *DNF2* and *SymCRK* (Bourcy *et al.*, 2013; Berrabah *et al.*, 2014*a*). These genes encode for a phosphatidyl inositol specific phospholipase C-like protein and a cysteine-rich receptor-like kinase respectively. Mutants altered in these genes develop nodules in which transcription of defence-related genes is induced and phenolic compounds accumulate (Bourcy *et al.*, 2013; Berrabah *et al.*, 2014*a*). Rhizobia viability is drastically reduced in these mutants maybe as a consequence of the observed defence-like reactions.

Interestingly, defences are not totally abolished in *M. truncatula* wild type (WT) nodules. Indeed, in indeterminate nodules (i.e. those harbouring a persistent meristem), legumes produce nodule-specific cysteine-rich (NCR) peptides that are defensin-like peptides (Mergaert *et al.*, 2003; Alunni *et al.*, 2007). NCR peptides are specifically produced in infected cells and have a toxic effect on bacteria and prevent their division. As a consequence, in *M. truncatula*, intracellular rhizobia (called bacteroids) are elongated bacteria, up to 10 µm long, that contain multiple genome copies, up to 24C (Mergaert *et al.*, 2006). NCR toxicity is partially countered by the bacterial BacA protein (Haag *et al.*, 2011), which is believed to act as a peptide importer. The bacterial *bacA* mutants are more susceptible to NCR peptides *in vitro* as well as *in planta* (Van de Velde *et al.*, 2010; Haag *et al.*, 2011). Indeed, once released into the plant cells the *bacA* mutant does not elongate as the WT bacteria and is rapidly killed (Haag *et al.*, 2011). Interestingly, the ability of the *bacA* mutant to survive within the plant cells is restored in the *M. truncatula dnf1* mutant (Haag *et al.*, 2011). *DNF1* is required for bacteroid differentiation and encodes a nodule-specific subunit of a signal peptidase necessary to address plant proteins (including NCR peptides) to the cellular compartments containing the bacteroids (Wang *et al.*, 2010). In addition to *DNF1*, three other genes (*RSD, DNF2* and *SymCRK*) are also required for bacteroid elongation in *M. truncatula* (Bourcy *et al.*, 2013; Sinharoy *et al.*, 2013; Berrabah *et al.*, 2014*a*). These three genes are specifically expressed in nodules and the symbiotic organs of the *rsd*, *dnf2* and *symCRK* mutants accumulate brown material (Bourcy *et al.*, 2013; Sinharoy *et al.*, 2013; Berrabah *et al.*, 2014*a*), reminiscent of defence reactions.

Defence-like reactions are not developed in *bacA*-triggered nodules indicating that the lack of bacteroid elongation and the lack of nitrogen fixation do not elicit *dnf2*- and *symCRK*-like defences (Berrabah *et al.*, 2014*b*). However, the *bacA* mutant is unable to colonize massively the plant cells and the question remains as to whether the lack of nitrogen fixation associated with a massive bacterial intracellular colonization could elicit the plant defences.

We aim to better understand the repression of defence-like reactions in nodules by identifying the factors involved in this process and by determining precisely the sequence of events that conduct defence development in the mutants. Herein we show that early bacterial death is associated with bacterial inability to fix nitrogen and that *RSD* is also required to prevent defence-like reactions in nodules. In addition, by combining the use of the *dnf2*, *symCRK* and *rsd* mutants together with various bacterial mutants, we define two steps in the symbiotic control of immunity after rhizobia internalization.

## Materials and methods

### Bacteria strains, plant lines, cultivation and inoculation methods

*Sinorhizobium meliloti* strain 1021 (Galibert *et al.*, 2001), *bacA* (Ferguson *et al.*, 2002), *nifH, nifA*, Sm2011, Rm41 (Kondorosi *et al.*, 1984), strain AK631 (*eps* ^*-*^) (Putnoky *et al.*, 1988), strain PP711(*kps* ^*-*^) (Becquart-de Kozak *et al.*, 1997) were cultivated in YEB medium (Krall *et al.*, 2002) for 24h at 30°C. *M. truncatula* ecotype R108, also referred to as *M. truncatula* ssp. *tricycla*, and its derived lines NF0737, i.e. *symCRK*, MS240, *dnf2-4*, NF11265, *rsd-1*, NF8776 and NF17452 (Tadege *et al.*, 2009; Pislariu *et al.*, 2012; Bourcy *et al.*, 2013; Sinharoy *et al.*, 2013; Berrabah *et al.*, 2014*a*; Cheng *et al.*, 2014) as well as *M. truncatula* cv. Jemalong, line A17 and the *dnf1* (Mitra and Long, 2004; Starker *et al.*, 2006; Wang *et al.*, 2010; Young *et al.*, 2011) derived line were cultivated as previously described (Berrabah *et al.*, 2014*a*). Briefly, seeds were scarified by incubation in sulphuric acid for 7 mins followed by 4 washing steps in sterile distilled water. After scarification, seeds were surface sterilized by incubation in 15ml of 0.3% chlorine followed by four washing steps in 50ml of sterile distilled water. Sterile seeds were then incubated at 4°C onto 1% agar (water) plates for 48h at 4°C. Seeds were then transferred to room temperature for 24 to 48h. After germination, seedlings were transferred onto buffered nodulation medium (BNM) (Ehrhardt *et al.*, 1992) supplemented with AVG 1 µM and immediately inoculated. Bacterial cells were washed with distilled sterile water before seedling inoculation. Optical densities of the bacterial cell suspension were adjusted to OD_600_=0.1 and 1ml of this suspension was used per 12×12cm plate containing eight seedlings. After 30 mins, liquid excess was removed by pipetting and the plates were sealed on three sides. Plants were cultivated in a growth chamber at 26°C at 60% humidity with a photoperiod of 16h/8h light/dark respectively.

### Molecular methods

Nodules were collected on plants with forceps and bistoury and immediately frozen in liquid nitrogen. Samples were reduced to powder by grinding material with steel beads in 2ml tubes using a Qiagen tissues lyser 2×30 s at 25 pulsations/second. RNAs were extracted using GeneJET Plant RNA Purification Mini kit (Thermo Scientific) as recommended by the manufacturer, RNA extracts were treated with Turbo DNAse ambion (Life Technology) to remove DNA traces. RNAs were reverse transcripted using SuperScriptII (Life Technology) as recommended by the manufacturer. qPCR was then carried out on Light Cycler 480 using the LightCycler 480 SYBER Green I (Roche) device as previously described (Gonzalez-Rizzo *et al.*, 2006). Samples were normalized using the constitutive *MtACTIN2* as a reference gene (Limpens *et al.*, 2005). Progeny of mutant lines altered in *DNF1* was genotyped using primers listed in Supplementary Table S1 following the method described in Ratet *et al.* (2010). Primers used are listed in Supplementary Table S1.

### Imaging and histological analysis

Entire nodules were imaged using Nikon macroscope AZ100. To produce sections, nodules were embedded in 6% agarose (water) and 70 µm sections were produced using vibratome VT1200S from Leica. To observe necrosis, nodule sections were also imaged with a macroscope AZ100. Sections mounted between slide and slipcover were bright light illuminated and observed with CIS illumination. For live and dead staining, we used the protocol previously described in Haag *et al.* (2011). Briefly, nodule sections were incubated in a 50mM Tris-HCl buffer (pH 7.2) containing 30 µM propidium iodide and 5 µM Cyto9 (Life Technology) for 20 mins. Stained sections were then mounted between slide and slipcover with a few Tris-HCl buffer drops and observed using a Leica SP8 confocal microscope.

For phenolic staining, a protocol derived from (Vasse *et al.*, 1993) was used. Briefly, nodule sections were fixed for 30 to 40 mins using potassium permanganate 0.02%. Sections were washed as necessary using PIPES 100mM (pH 7.2) buffer to remove precipitate. Phenolics were then stained with methylene blue 0.01% for 10 to 15 mins and nodule sections were washed in 6.1% chlorine solution prior observation.

### Acetylene reduction assay

An acetylene reduction assay was conducted as previously described (Berrabah *et al*., 2014*a*, *b*). The method is derived from the one described in Koch and Evans (1966). Briefly, entire plants were individually incubated in 10ml vials containing air supplemented with 250 µl acetylene. After at least 1h at room temperature, 200 µl of the gas phase were analysed by gas chromatography coupled with flame ionization detection.

### *In situ* hybridization

*In situ* hybridizations followed the protocol described in Boualem *et al.* (2008). Twenty-one dpi nodules of *M. truncatula* cultivated *in vitro* on BNM-agar were analysed using RNA probes labelled with dioxygenin and revealed using antibodies coupled to alkaline phosphatase. The anti-sense probe was designed on extracellular cysteine-rich *SymCRK* domain to specifically target the *SymCRK* transcript. The sense probe was used as a negative control; probes were synthethized using T7/SP6 primers after target sequence cloning in the pGEMT easy vector. The primers used to amplify the target region are listed in Supplementary Table S1.

## Results

### The *nifA* fix^-^ mutant undergoes bacterial death that is not associated with *symCRK*/*dnf2*-like defence reactions

**Fig. 1.**
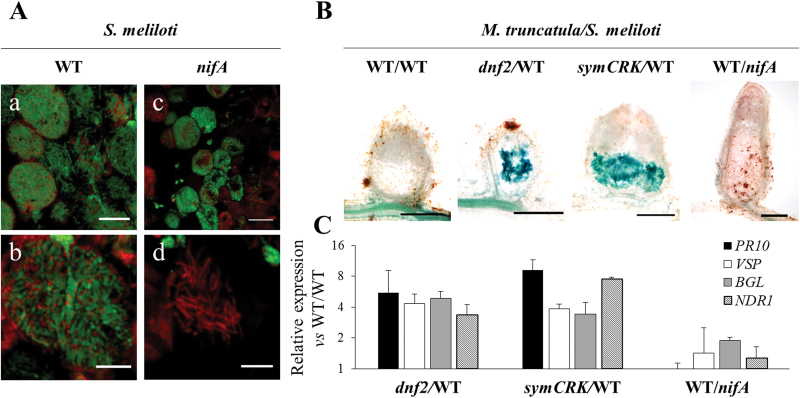
Mutation in *nifA* elicits early bacterial death that is not mediated by *symCRK*/*dnf2*-dependant defence reactions. (A): a–d: On these sections of 26dpi nodules imaged using a confocal microscope after live/dead staining assay, the early death of the *nifA* mutant within the plant cell is observable. Green and red stain alive and dead bacteria respectively. a and b represent WT bacteria induced nodules, c and d: *nifA* induced nodules, note that like the WT bacteria, the *nifA* mutant undergoes terminal differentiation, scale bars correspond to, 20µm (a, c) and 10µm (b, d). (B): *nifA* mutant does not elicit the accumulation of phenolics in nodules. Sections of WT plant nodules induced by WT bacteria or by *nifA* mutant as well as *dnf2* and *symCRK* nodules induced by WT bacteria were stained with methylene blue to reveal phenolics in blue. Scale bars correspond to 500 µm. (C): *PR10*, *VSP*, *BGL* and *NDR1* defence-related genes are not induced in *nifA-*triggered nodules. In contrast these defence-related genes are induced in *dnf2* and *symCRK* nodules as revealed by RT-qPCR analysis. Results are expressed as induction fold vs R108 nodules induced by WT bacteria after normalization using *Mtactin2* constitutive gene as an internal standard.

Using the bacterial *bacA* mutant that is unable to differentiate elongated bacteroids, we previously showed that lack of bacteroid differentiation does not elicit expression of defence genes or phenolics accumulation that are observed in the *dnf2* and the *symCRK* nodules (Berrabah *et al.*, 2014*b*). Nevertheless, the *bacA* mutant is hypersensitive to NCR peptides and this sensitivity does not allow massive colonization of the plant cells (Haag *et al.*, 2011). In order to determine if a massive colonization by ineffective bacteria triggers defence reactions, *M. truncatula* WT plants were inoculated with the *S. meliloti nifA* mutant. NifA is a key regulator of the nitrogenase synthesis and the corresponding mutant does not fix nitrogen (Zimmerman *et al.*, 1983; Dixon and Kahn, 2004). Nodules induced by *nifA* are infected and bacteroids clearly undergo terminal differentiation (Fig. 1). However, in contrast to the WT, the mutant bacteria prematurely died as revealed by the live/dead assay that stains viable bacteria green and dead bacteria red (Fig. 1). The bacterial death occurs only after bacteroid elongation in the *nifA* mutant (Supplementary Fig. S1). Beyond genes responsible for nitrogenase synthesis, NifA regulates additional genes (Bobik *et al.*, 2006) and the *nifA* mutant displays pleiotropic phenotypes (Gong *et al.*, 2007). To determine whether *nifA* bacteroid death is caused by the lack of nitrogen fixation or to another *nifA*-regulated process, *nifH* bacteroid viability was also studied; *nifH* encodes a subunit of the nitrogenase. Like for the *nifA* mutant, bacteroid death was observed for elongated *nifH* bacteroids (Supplementary Fig. S1) suggesting that lack of nitrogen fixation is responsible for the death of *nifA* and *nifH* elongated bacteroids. In order to determine if defence reactions such as those observed in the nodules of the *symCRK* and the *dnf2* mutants could be responsible for *nifA* bacterial death, the induction of these defences was evaluated by RT-qPCR and histological approaches. In contrast to the *dnf2* and *symCRK* mutant nodules, the expression of the defence-related genes *PR10*, *VSP*, *BGL* and *NDR1* was not induced in the *nifA*-triggered nodules, which also do not accumulate phenolics (Fig. 1). In agreement of a role of *nifA* after *DNF2* and *SymCRK*, mutation in the *nifA* gene does not prevent the development of defence-like reactions in *dnf2* and *symCRK* nodules (Supplementary Fig. S2). Together these results suggest that lack of nitrogen fixation induces early death of the bacteria. Furthermore this bacterial death is not associated with defence-like reactions observed in the *dnf2* and *symCRK* mutants (Fig. 1).

### *DNF2, bacA* and *SymCRK* act successively during the symbiotic process

**Fig. 2.**
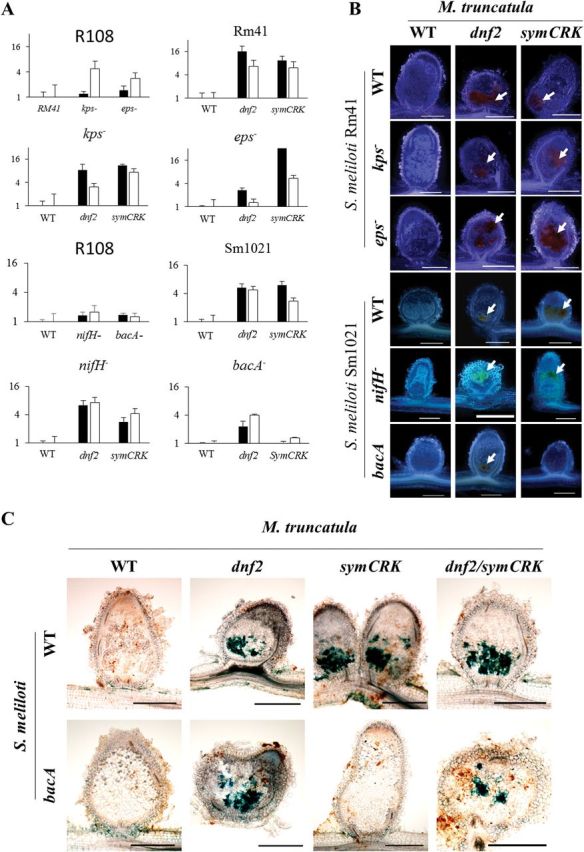
*DNF2*, *bacA* and *SymCRK* act successively during the symbiotic process. (A) Development of defence reactions was investigated in nodules by evaluating the expression of defence-related genes *PR10* and *NDR1*. The four top sub-panels correspond to plants inoculated with Rm41 WT or the corresponding mutant strains. The four bottom sub-panels correspond to plants inoculated with Sm1021 WT or the corresponding mutant strains. The top left sub-panel of each four sub-panels corresponds to gene expression in the R108 WT plants inoculated by the WT bacteria or its mutant derivatives. The three other sub-panels correspond to gene expression in the R108 WT or *dnf2* or *symCRK* mutants inoculated by different bacteria. (B) The development of defence reactions was investigated in nodules by examination of necrotic zone in nodule section. Columns represent the plant genotypes and rows the bacterial genotypes. Arrows in (B) point to the necrotic zones. (C) Accumulation of phenolic compounds is revealed by the blue color on nodule sections stained with methylene blue. B, C: scale bars correspond to 500 µm.

In order to get an insight into the development of the symbiotic process and the timing of immunity suppression during symbiosis, nodulation tests were conducted using *dnf2* and *symCRK* mutants in combination with bacterial mutants altered in surface components or in the symbiotic process. Defence-like reactions were evaluated 14 days after inoculation in the nodules induced by *S. meliloti* strain Rm41 eps^-^ and kps^-^ mutants (altered in exopolysaccharide and capsular polysaccharide respectively) as well as *S. meliloti* Sm1021 *bacA*, and *nifH* mutants. Defence reactions were monitored by following the expression of the *NDR1* and *PR10* genes using RT-qPCR. In addition, nodule sections were observed under bright field illumination to reveal the presence of necrotic zones accumulating phenolics. The *eps* and *kps* mutants elicit a moderate induction of the expression of *NDR1* and *PR10* genes in nodules of the WT plants (Fig. 2A). These inductions were not associated with the development of necrotic zones in the WT plant nodules (Fig. 2B). In contrast, the expression of defence-related genes was observed when these bacterial mutants were nodulating *dnf2* and *symCRK* plant mutants and this was associated to the presence of necrotic zones (Fig. 2B). In nodules developed on WT plants inoculated by Sm1021 or its *nifH* or *bacA* mutant derivatives, the *NDR1* and *PR10* genes are not induced (Fig. 2A) and these nodules do not accumulate phenolics. In contrast to the WT plants, *dnf2* and *symCRK* nodules display necrosis and induction of the defence markers with these strains, except for *symCRK* inoculated with the *bacA* mutant; in this plant/bacteria mutant combination the expression of defence-related genes and nodule necrosis were not observed (Fig. 2A, B). This absence of defence reactions in *symCRK/bacA* nodules was further confirmed in 21 and 27 day-old nodules (Fig. 2C, Supplementary Fig. S3). However, acetylene reduction assays indicate that nitrogen fixation was not restored in the *symCRK*/*bacA* nodules (Supplementary Fig. S4). Furthermore, despite the reduction in the expression of late *NCR* genes (Berrabah *et al.*, 2014*a*) (Supplementary Fig. S5) and the absence of defence-like reactions in the *symCRK* mutant upon *bacA* infection, the *bacA* mutant viability was not restored in these *symCRK/bacA* nodules (Supplementary Fig. S6) contrasting with what was observed in *dnf1*/*bacA* nodules (Haag *et al.*, 2011).

The double mutant *dnf2symCRK* was analysed with respect to defence development in nodules elicited with WT bacteria or *bacA* mutant strain. We did not observe any additive effect of the mutation in the double mutant nodules upon inoculation with the WT bacteria (Fig. 2C, Supplementary Figs S3 and S6). When inoculated with the *bacA* mutant, defence reactions were observed in the *dnf2symCRK* nodules but not on the *symCRK* mutant (Fig. 2C, Supplementary Fig. S3). These results thus indicate that the double mutant behaves as the *dnf2* mutant in terms of defence reactions and that *DNF2* is epistatic to *SymCRK* in the *M. truncatula* R108 background. Together, these results suggest that *DNF2*, *bacA* and *SymCRK* act successively during the symbiotic process.

### *SymCRK* correct expression requires initiation of bacteroids differentiation

**Fig. 3.**
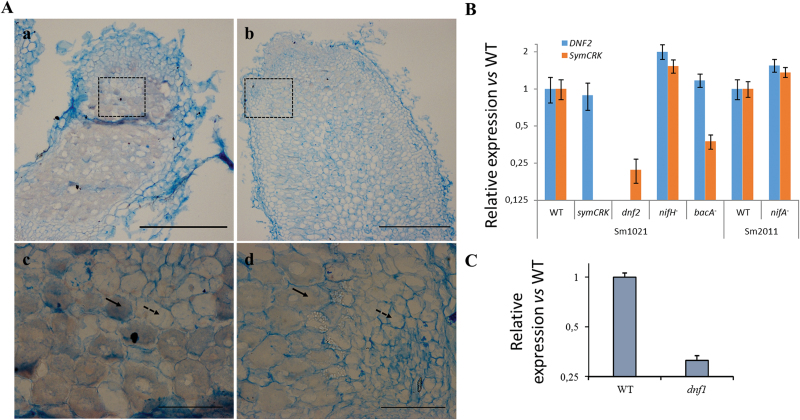
*SymCRK* is expressed in infected cells. (A) Expression of *SymCRK* was investigated using *in situ* hybridization and (B, C) RT-qPCR. Using antisense probe (a, c), signal was detected in infected cells (indicated with plain arrows) of the nitrogen fixation zone and not in uninfected cells (indicated with dashed arrows); sense probe was used as a control of specificity (b, d). c, d represent magnification of the zones delimited by a dashed rectangle in a and b. Scale bars represent 300 and 30 µm in whole nodule sections and in enlargement respectively. (B) RT-qPCR analyses revealed that *SymCRK* expression is strongly reduced in nodules of the *dnf2* mutant. In contrast, the expression of *DNF2* is not altered in the *symCRK* nodules. Also, expression of *SymCRK* was strongly reduced in the *bacA* induced nodules (B) as well as in the 14dpi nodules of the *dnf1* mutant (C). B, C: error bars represent standard errors of three biological experiments with two technical replicates.

In order to improve our understanding of the *SymCRK* gene function, its expression pattern inside nodules was studied using *in situ* hybridization in WT nodules. This experiment (Fig. 3A) suggests that *SymCRK* is specifically expressed in infected cells in agreement with its timing of expression (Berrabah *et al*., 2014*a*; see also below). In order to define more precisely the role of *SymCRK* and *DNF2* during the symbiotic process, we investigated the expression of the two genes in *M. truncatula* nodules induced by the *S. meliloti nifA* and *nifH* mutants altered in the production of nitrogenase (for review, see Dixon and Kahn, 2004) as well as by the *bacA* mutant. In nodules induced by the *nifA* and *nifH* bacterial mutants (Fig. 3B), expression of the two symbiotic genes *SymCRK* and *DNF2* was not altered, indicating that nitrogen fixation is not necessary for their expression. *DNF2* transcript accumulation is not modified in nodules induced by the *bacA* mutant or in *symCRK* mutant nodules induced by WT bacteria placing *DNF2* upstream or in a parallel pathway to *BacA* and *SymCRK*. In contrast, the level of *SymCRK* transcripts was reduced in WT nodules induced by the *bacA* mutant or in *dnf2* mutant nodules induced by WT bacteria, in agreement with the data shown above, indicating that *SymCRK* act downstream of *DNF2* and *bacA* during the symbiotic process. As mentioned previously, bacteroids do not differentiate and are not maintained viable in both the *dnf2* and the *bacA* nodules. In contrast, in the *dnf1M. truncatula* mutant, bacteria do not undergo terminal differentiation but bacteroids remain viable during colonization (Haag *et al.*, 2011). Thus, this mutant constitutes a good tool to discriminate whether the colonization defect and/or the differentiation defect is responsible for incorrect *symCRK* expression. We have used two *Tnt1* insertion mutant lines for *DNF1* (NF8776 and NF17452) in the *M. truncatula* R108 background (in which the *dnf2* and *symCRK* mutants used in this study were generated). In these lines the *Tnt1* retrotransposon is inserted in the first intron 131bp and 324bp after the predicted start codon. However, we could not observe any symbiotic phenotypes in the progeny, possibly as a result of the insertion in the intron or of the absence of homozygous mutant (Supplementary Fig. S7). The expression of *SymCRK* was thus studied in the only *dnf1* available mutant (generated in the A17 background). This experiment shows that *SymCRK* expression was reduced in the *dnf1* mutant, suggesting that not only bacterial colonization but also bacteroid differentiation is required for *SymCRK* expression (Fig. 3C).

### *RSD* represses defence-like reactions and act downstream of *bacA* and *DNF2*

**Fig. 4.**
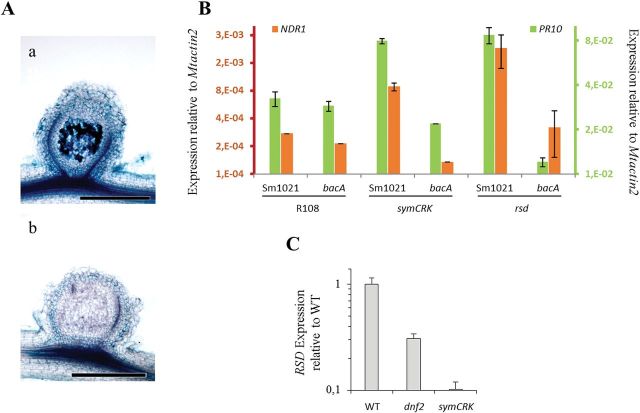
*RSD* is required to suppress immunity in nodules and act downstream of *bacA*. (Aa) Upon inoculation with the WT *Sinorhizobium meliloti*, the *rsd-1* mutant nodules (32dpi) accumulate phenolics as revealed by methylene blue staining of nodules sections. (Ab) In contrast, *bacA* triggered nodules do not accumulate phenolics; scale bars represent 500 µm. (B) In agreement, RT-qPCR analyses indicate that defences are activated in *rsd* nodules induced by the WT bacteria but not in those triggered by the *bacA* mutant. (C) *RSD* expression is strongly reduced in *dnf2* and *symCRK* mutant nodules. B, C: error bars represent standard errors.

Like *dnf2* and *symCRK*, *rsd* displays necrotic nodules in which bacteroids do not undergo terminal differentiation (Sinharoy *et al.*, 2013). In order to determine if *rsd* also shares the defence-like reactions observed in the *dnf2* and *symCRK* mutants, induction of defence-related genes was evaluated in *rsd-1* nodules. Expression of the *PR10* and *NDR1* genes was evaluated by RT-qPCR and was found to be induced in *rsd-1* as compared to WT nodules (Fig. 4). In addition, presence of phenolic compounds was detected in *rsd-1* nodules (Fig. 4) indicating that *rsd* develops defence-like reactions similar to those of *symCRK* and *dnf2*. In order to position *RSD* with respect to *SymCRK* and *DNF2* during the development of the symbiosis, we analysed the expression of *RSD* in the *dnf2* and in the *symCRK* backgrounds. *RSD* expression is strongly reduced in both mutants (Fig. 4C) suggesting a role for *RSD* downstream of *DNF2 and SymCRK*.

In order to determine whether the development of defence-like reactions in the *rsd-1* mutant is dependent on bacterial invasion and differentiation, we evaluated defences in this mutant upon nodulation with the *bacA* mutant. In contrast to *dnf2* and, similarly to *symCRK*, the *rsd* mutant does not accumulate phenolics upon infection with *bacA* (Fig. 4A). In agreement with these results, defence-related gene expression was strongly reduced in *rsd*/*bacA* nodules as compared to *rsd* nodules triggered by WT bacteria (Fig. 4B). Thus, similar to what is observed in the *symCRK* mutant, development of defence-like reactions in *rsd* relies on the presence of a functional *bacA*.

## Discussion

Herein we have investigated the sequence leading to the symbiotic suppression of immunity in legume nodules after rhizobia internalization within the plant cells (chronic infection). We showed that the bacterial mutants *nifA* and *nifH* died prematurely within the plant cells despite that defence reactions similar to those observed in *symCRK* and *dnf2* nodules were not detected in these nodules. In addition, we showed that *DNF2* and *SymCRK* act successively during the symbiotic process and that *bacA* is required after *DNF2* and before *SymCRK*. Furthermore, our data indicate that *rsd-1* behave like the *symCRK* mutant and acts after *bacA*. These results are in agreement with the observation that *SymCRK* and *RSD* expression is reduced in WT plants nodulated with *bacA,* a condition in which expression of *DNF2* is not modified (Fig. 3B; Sinharoy *et al.*, 2013). In addition, results obtained with the *dnf1* mutant suggest that the initiation of bacteroid differentiation rather than the massive intracellular colonization is important for *SymCRK* activity. Interestingly, in the distant phylogenetic system, soybean/*Bradyrhizobium japonicum*, the *nifA* mutant triggers nodules displaying dark brown zones of necrotic appearance (Fischer *et al.*, 1986) reminiscent of *symCRK*/*dnf2*-like defences. Altogether our results indicate that multiple steps are required for the symbiotic suppression of immunity after bacterial internalization. Furthermore the present study indicates that at least two types of defence can be the cause of bacterial death in the plant cells, one associated with nodule necrosis and induction of *PR10* and *NDR1* defence genes and another observed upon *nifA-* or *nifH*-triggered nodulation.

**Fig. 5.**
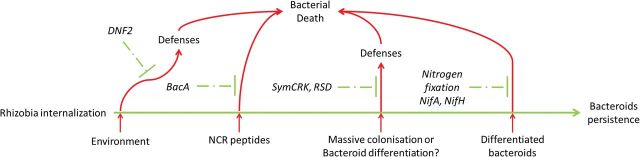
Bacteroid death is prevented by multiple actors acting successively. *DNF2* is the earliest actor identified as required for symbiotic suppression of immunity at the intracellular stage of the symbiosis. Its requirement is determined by environmental conditions that influence the development of defence-like reactions in nodules (Berrabah *et al.*, 2014*a*). After *DNF2*, the *bacA* bacterial gene prevents the NCR triggered bacteroid death (Haag *et al.*, 2011). Later, *SymCRK* and *RSD* prevent defence-like reactions possibly triggered by massive intracellular invasion or initiation of bacteroid differentiation. Finally, nitrogen fixation is required to prevent the death of elongated bacteroids.

Based on our results, we propose a model focused on the intracellular stage of symbiosis, which describes the actors preventing bacteroid death (Fig. 5). In this model, *DNF2* is the earliest actor identified as required for symbiotic suppression of immunity. Its requirement is determined by environmental conditions that influence the development of defence-like reactions in nodules (Berrabah *et al.*, 2014*b*). After *DNF2*, the *bacA* bacterial gene prevents the killing activity of NCR peptides before *SymCRK* and *RSD* prevent defence-like reactions possibly triggered by massive intracellular invasion or initiation of bacteroid differentiation. Finally, the production of nitrogenase prevents bacteroid death. In order to add more actors to this model, necrotic mutants (such as those described in Pislariu *et al.*, 2012; Domonkos *et al.*, 2013) will probably represent good tools. Such mutants could in future studies be classified according to their behaviour upon inoculation in combination with bacterial symbiotic mutants (amongst which is *bacA*) but also based on their symbiotic properties using *dnf2* permissive media (Berrabah *et al.*, 2014*b*).

Immunity suppression is not only required for the intracellular stage of the symbiosis; various actors have been identified or proposed to be important to prevent plant defences during the infection process. For example the nodulation (Nod) factors might participate in the suppression of plant immunity triggered by microbial motifs (Liang *et al.*, 2013). In agreement with this novel Nod factor’s role, the type three secretion system (often used by phytopathogenic bacteria to suppress plant immunity) of *Bradyrhizobium elkanii* can bypass the requirement of Nod factors for the nodulation of soybean (Okazaki *et al.*, 2013). Also, two soybean resistance genes modify the rhizobial host range of the plant (Yang *et al.*, 2010) and exopolysaccharides were shown to be important to reduce the induction of defence-related genes at the early stages of the symbiosis (Jones *et al.*, 2008). Finally, defence-related phytohormones also interfere with the nodulation process (Penmetsa and Cook, 1997; Stacey *et al.*, 2006; Sun *et al.*, 2006). These data indicate that suppression of immunity during the early steps of the symbiotic process is required for nodulation. In agreement with this, the expression of a *PR10* gene is drastically reduced in bumps (young nodules) and mature nodules as compared to roots. We showed that *DNF2* prevents defence-like reactions in nodules only after bacteria internalization (Bourcy *et al.*, 2013) and that in bumps, the expression of the *PR10* gene is similar in the *dnf2* and WT nodules (Bourcy *et al.*, 2013). These results indicate that a mechanism distinct from *DNF2* and *SymCRK* suppresses immunity in early developing nodules and reinforce the hypothesis of the existence of multiple steps in the suppression of plant immunity during the symbiotic process. It is now a challenge to gather the identified actors of the symbiotic suppression of immunity within an integrated model.

## Supplementary data

Supplementary data are available at *JXB* online.

Supplementary Fig. S1. Lack of nitrogen fixation triggers bacterial death after bacteroid elongation.

Supplementary Fig. S2. *dnf2* and *symCRK* act before *nifA*.

Supplementary Fig. S3. Defence reactions are abolished in the *symCRK bacA* nodules.

Supplementary Fig. S4. Nitrogen fixation is not restored in *symCRK*/*bacA* nodules.

Supplementary Fig. S5. NCR99 expression is reduced in *bacA*-triggered nodules of the *dnf2* and *symCRK* mutants.

Supplementary Fig. S6. *bacA* viability is not restored in the *symCRK* mutant.

Supplementary Fig. S7. *DNF1* gene structure (based on MTR_3g027890 sequence) and position of the *Tnt1* insertions present in mutant lines NF8776 and NF17452.

Supplementary Table S1. List of primers used during this study.

Supplementary Data
