# Supplementary material for: Multiple steps control immunity during the intracellular accommodation of rhizobia
Source: J Exp Bot. 2015 Feb 14;66(7):1977–85. doi: 10.1093/jxb/eru545 (PMC4378630; doi:10.1093/jxb/eru545)
Supplement: Supplementary Data [file supp_66_7_1977__index.html]

Multiple steps control immunity during the intracellular accommodation of rhizobia — Supplementary Data 

# Multiple steps control immunity during the intracellular accommodation of rhizobia

## Supplementary Data

Data files

**Files in this Data Supplement:**

- Supplementary Data - Supplementary Data
