# Supplementary material for: Multiple steps control immunity during the intracellular accommodation of rhizobia
Source: J Exp Bot. 2015 Feb 14;66(7):1977–85. doi: 10.1093/jxb/eru545 (PMC4378630; doi:10.1093/jxb/eru545)
Supplement: Supplementary Data [file supp_eru545_eru545_Supplementary_Data_corrected_ref.doc]

**Multiple steps control immunity during the intracellular accommodation of rhizobia.**

**Fathi Berrabah, Pascal Ratet, Benjamin Gourion**

**Supplementary data:**

**Supplementary Fig. S1**. Lack of nitrogen fixation triggers bacterial death after bacteroid elongation. (A) Live/dead assay, which stains alive and dead bacteria in green and red respectively, was performed on 17 dpi nodule sections before observation with confocal microscope. After bacteroid elongation the *nifA* and *nifH* mutants encounter death in nodule cells. Bars, 30 µm. Meristematic zones are delimited by dot lines. Asterisks and the associated distance expressed as µm indicate the distance to the meristem. (B) Percentages of dead bacteria in different regions of the nodules (indicated by their distance to the meristem). Green and red bacteroids were counted in every regions (n>=60). The data represent the results of two independant experiments.


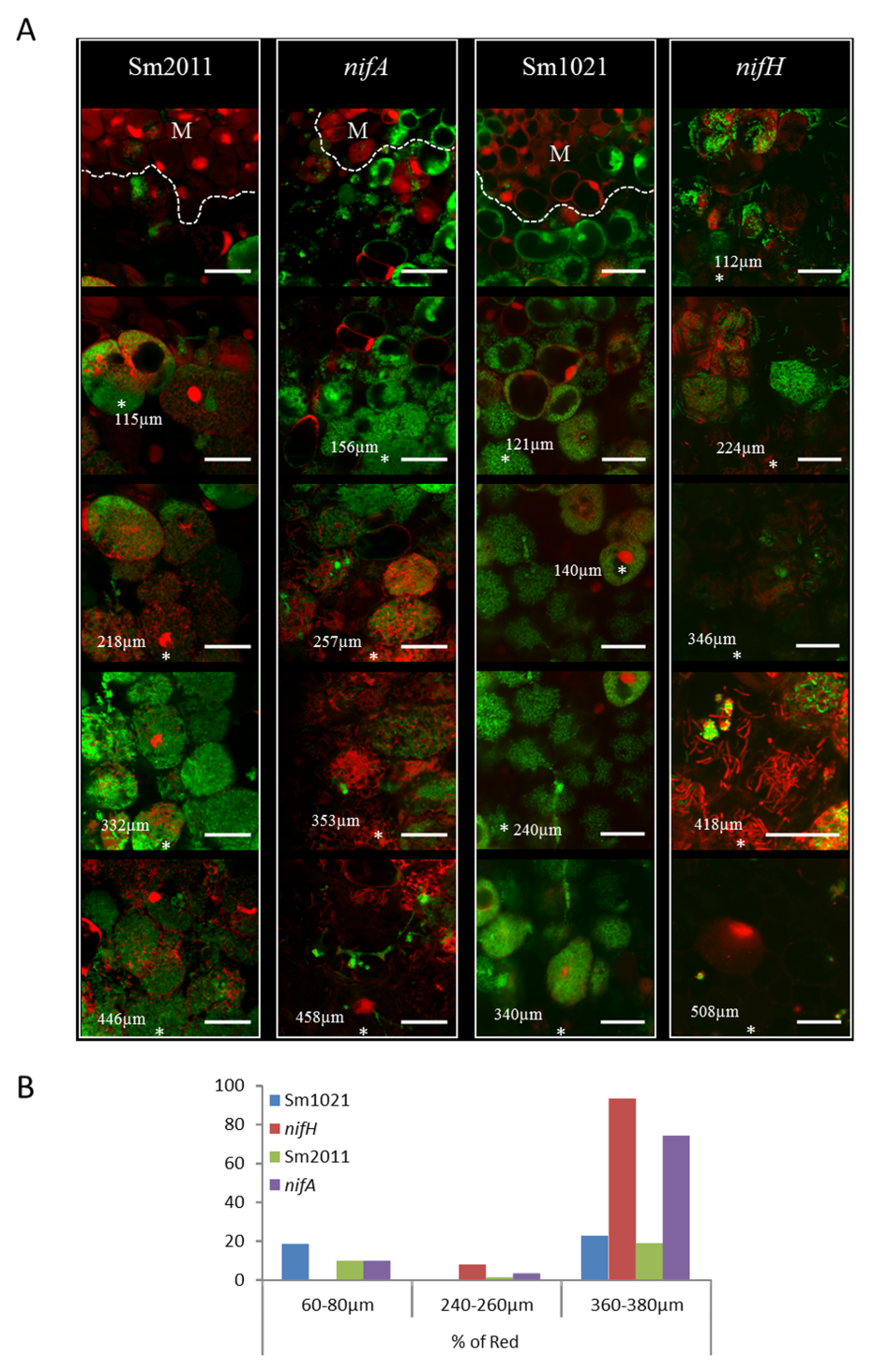


**Supplementary Fig. S2.** *dnf2* and *symCRK* act before *nifA*. Methylene blue staining of nodule sections after potassium permanganate fixation reveals the accumulation of phenolics in blue in nodules formed by the *dnf2* and the *symCRK* mutants. Bars, 500 µm.


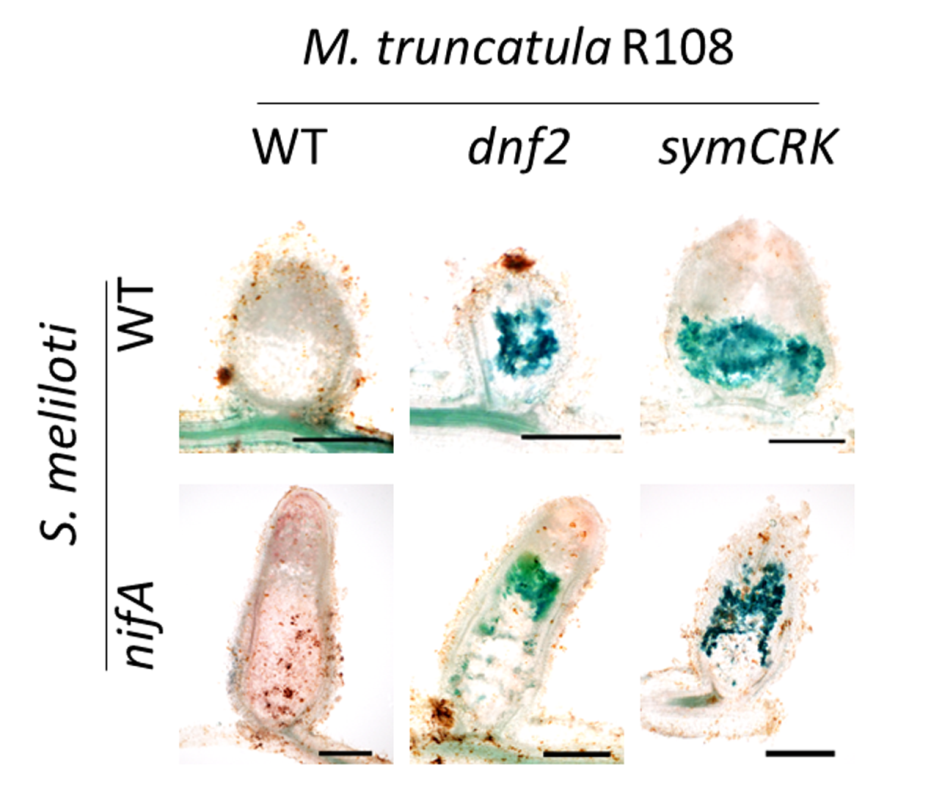


**Supplementary Fig. S3**. Defense reactions are abolished in the *symCRK bacA* nodules. Expression of *PR10* and *NDR1* was determined by RT-qPCR on RNAs extracted from the indicated nodule combinations (27 days after inoculation). In contrast to the *dnf2* and to the *dnf2symCRK* nodules, the *symCRK* nodules do not develop defence reactions upon inoculation with the bacterial *bacA* mutant, indicating that *dnf2*, *bacA* and *SymCRK* act successively during the symbiotic process. Error bars correspond to standard errors. Three biological repetitions with two technical replicates were made.


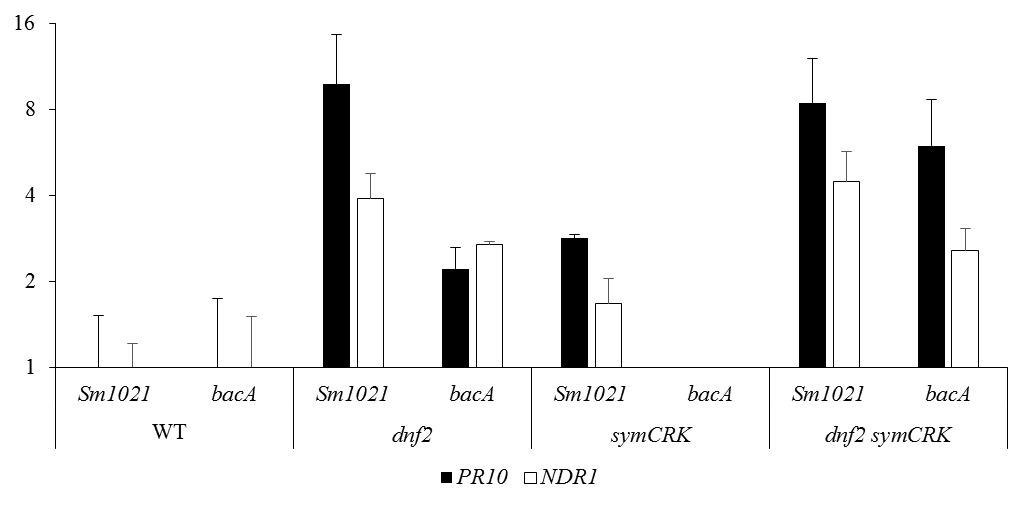


**Supplementary Fig. S4:** Nitrogen fixation is not restored in *symCRK*/*bacA* nodules. Acetylene reduction assays were conducted on *M. truncatula* WT, *dnf2* and *symCRK* plants nodulated with *S. meliloti* WT strain and with a *bacA* mutant. Measurements were taken on single plants and activities expressed relative to the WT plants nodulated with WT bacteria. Error bars represent standard errors. Three biological repetitions with three technical replicates were made.


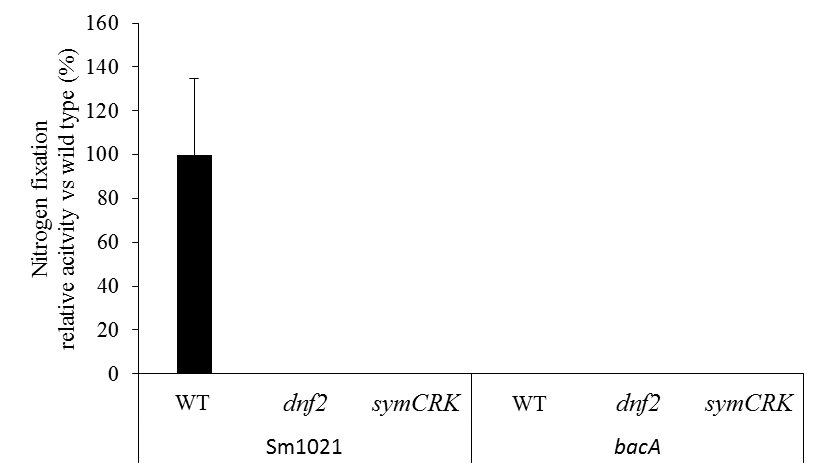


**Supplementary Fig. S5.** NCR99 expression is reduced in *bacA*-triggered nodules of the *dnf2* and *symCRK* mutants. Accumulation of the early (NCR121) and late (NCR99) *NCR* genes transcript were evaluated using RT-qPCR. cDNA were prepared from WT, *dnf2* and *symCRK* 27 dpi nodules triggered by *Sinorhizobium meliloti* WT and *bacA* strains. Transcript abundance is expressed relative to the WT nodules trigged by WT bacteria after normalization with an actin constitutive gene. Error bars represent standard errors. Three biological repetitions with two technical replicates were made for each type of sample.

***
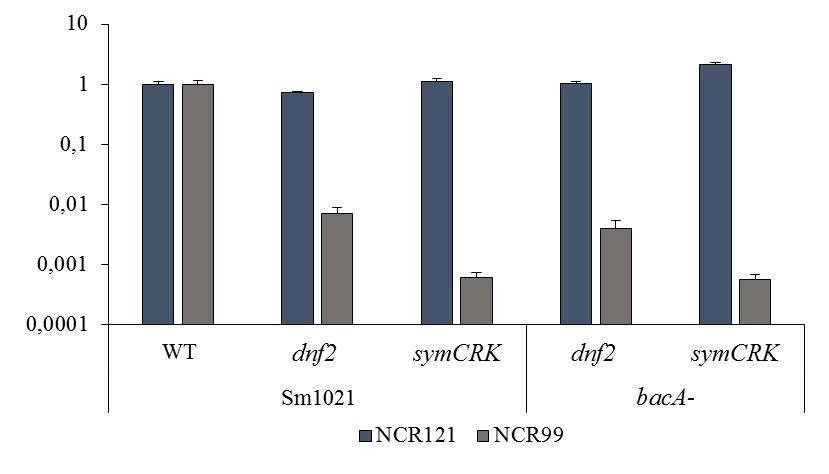
***

**Supplementary Fig. S6.** *bacA* viability is not restored in the *symCRK* mutant. Live/dead assay, which stains alive and dead bacteria in green and red respectively, was performed on 21 dpi nodule sections before observation with confocal microscope. The *bacA* mutant viability is not restored in the *symCRK* nodules. Bars, 500 µm for whole nodule section images and 30 µm for enlargements.


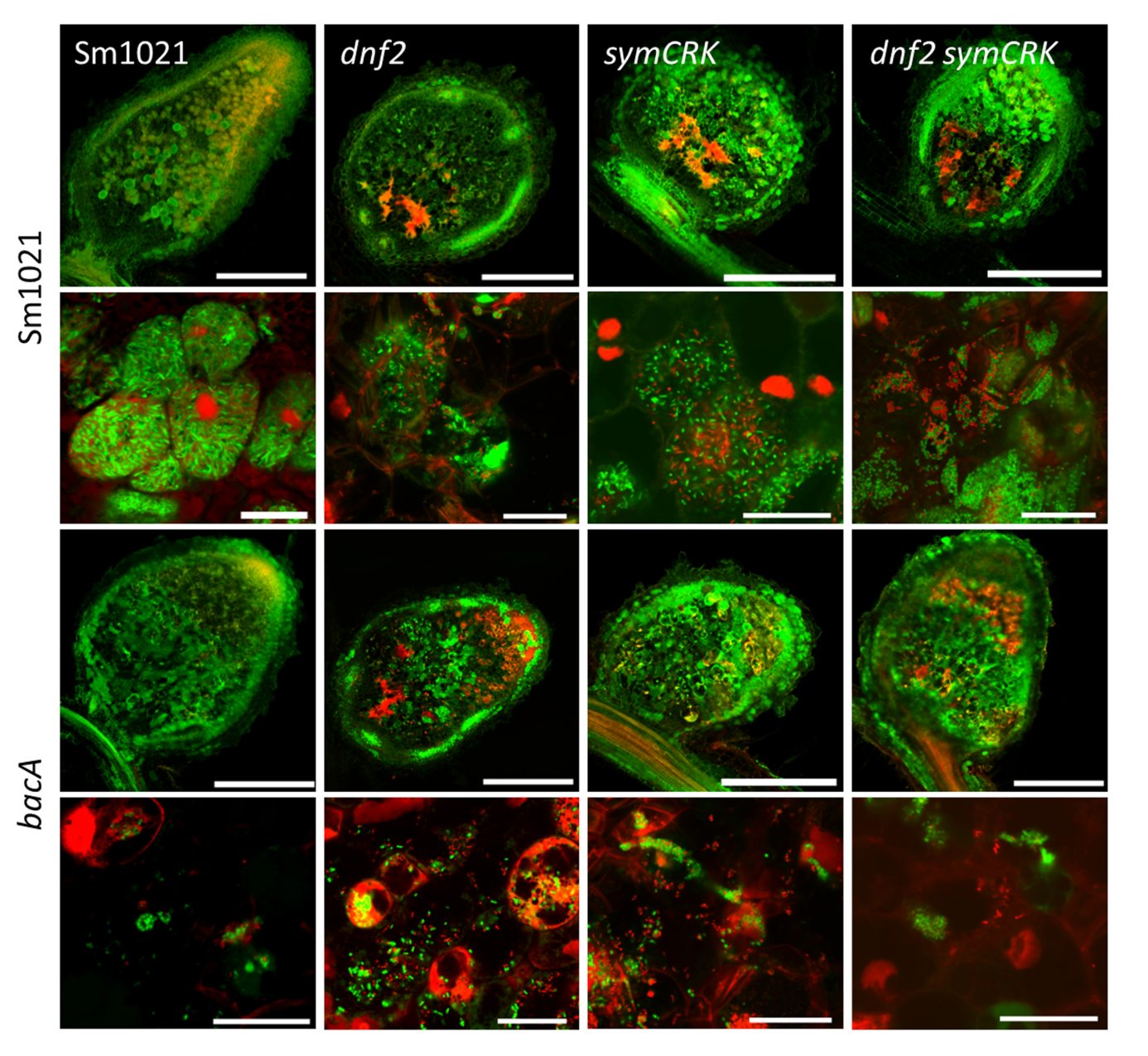


**Supplementary Fig. S7.** (A) *DNF1* gene structure (based on MTR_3g027890 sequence) and position of the *Tnt1* insertions present in mutant lines NF8776 and NF17452. White and red regions correspond to intronic and exonic sequences respectively. The position in bp of the stop codon relative to the start codon (AUG) is also indicated. (B) Genotyping and phenotyping of candidate *dnf1* mutant lines in the *M. truncatula* R108 ecotype. Plants were cultivated in sand/perlite substrate and inoculated with *Sinorhizobium meliloti* strain Rm41. Wild type (WT), heterozygous (het) and mutant (mut) genotypes were determined by PCR (? indicates WT or Het). Phenotype was determined six weeks after inoculation based on nodule colour.


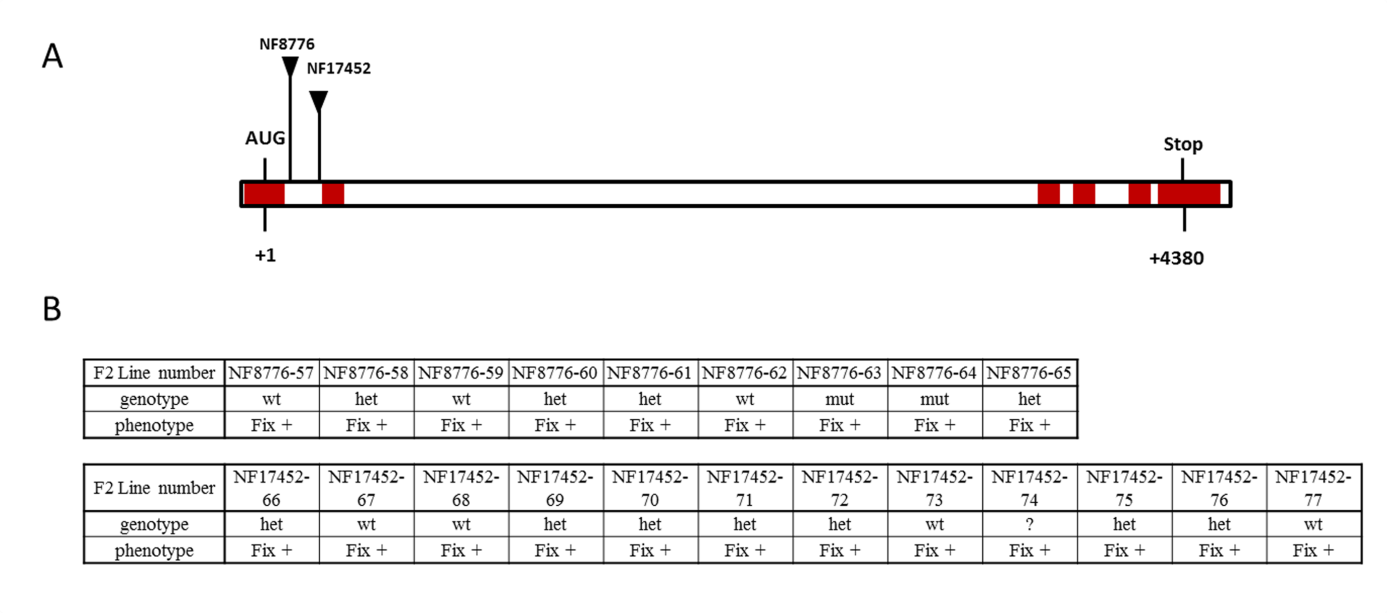


**Supplementary Table S1.**

List of primers used during this study

**Berrabah F, Bourcy M, Cayrel A, Eschstruth A, Mondy S, Ratet P, Gourion B**. 2014a. Growth conditions determine the DNF2 requirement for symbiosis. *PLoS One* **9**, e91866.

**Berrabah F, Bourcy M, Eschstruth A, Cayrel A, Guefrachi I, Mergaert P, Wen J, Jean V, Mysore KS, Gourion B, Ratet P**. 2014b. A nonRD receptor-like kinase prevents nodule early senescence and defense-like reactions during symbiosis. *New Phytologist*.

**Bourcy M, Brocard L, Pislariu CI, Cosson V, Mergaert P, Tadege M, Mysore KS, Udvardi MK, Gourion B, Ratet P**. 2013. *Medicago truncatula* DNF2 is a PI-PLC-XD-containing protein required for bacteroid persistence and prevention of nodule early senescence and defense-like reactions. *New Phytologist* **197**, 1250-1261.

**Gao LL, Anderson JP, Klingler JP, Nair RM, Edwards OR, Singh KB**. 2007. Involvement of the octadecanoid pathway in bluegreen aphid resistance in *Medicago truncatula*. *Mol Plant Microbe Interact* **20**, 82-93.

**Guefrachi I, Nagymihaly M, Pislariu CI, Van de Velde W, Ratet P, Mars M, Udvardi MK, Kondorosi E, Mergaert P, Alunni B**. 2014. Extreme specificity of NCR gene expression in *Medicago truncatula*. *BMC Genomics* **15**, 712.

**Limpens E, Mirabella R, Fedorova E, Franken C, Franssen H, Bisseling T, Geurts R**. 2005. Formation of organelle-like N2-fixing symbiosomes in legume root nodules is controlled by DMI2. *Proc Natl Acad Sci U S A* **102**, 10375-10380.

**Ratet P, Wen J, Cosson V, Tadege M, Mysore KS**. 2010. *Tnt1* Induced Mutations in *Medicago*: Characterization and Applications. *The Handbook of Plant Mutation Screening*: Wiley-VCH Verlag GmbH & Co. KGaA, 83-99.

**Samac DA, Penuela S, Schnurr JA, Hunt EN, Foster-Hartnett D, Vandenbosch KA, Gantt JS**. 2011. Expression of coordinately regulated defence response genes and analysis of their role in disease resistance in *Medicago truncatula*. *Mol Plant Pathol* **12**, 786-798.

**Sinharoy S, Torres-Jerez I, Bandyopadhyay K, Kereszt A, Pislariu CI, Nakashima J, Benedito VA, Kondorosi E, Udvardi MK**. 2013. The C2H2 transcription factor regulator of symbiosome differentiation represses transcription of the secretory pathway gene *VAMP721a* and promotes symbiosome development in *Medicago truncatula*. *Plant Cell* **25**, 3584-3601.
